# Supplementary material for: Consequences of Nephrotic Proteinuria and Nephrotic Syndrome after Kidney Transplant
Source: Biomedicines. 2024 Mar 30;12(4):767. doi: 10.3390/biomedicines12040767 (PMC11048274; doi:10.3390/biomedicines12040767)
Supplement: Supplementary file 1 [file biomedicines-12-00767-s001.zip › biomedicines-2915710-supplementary.pdf]

Figure S1

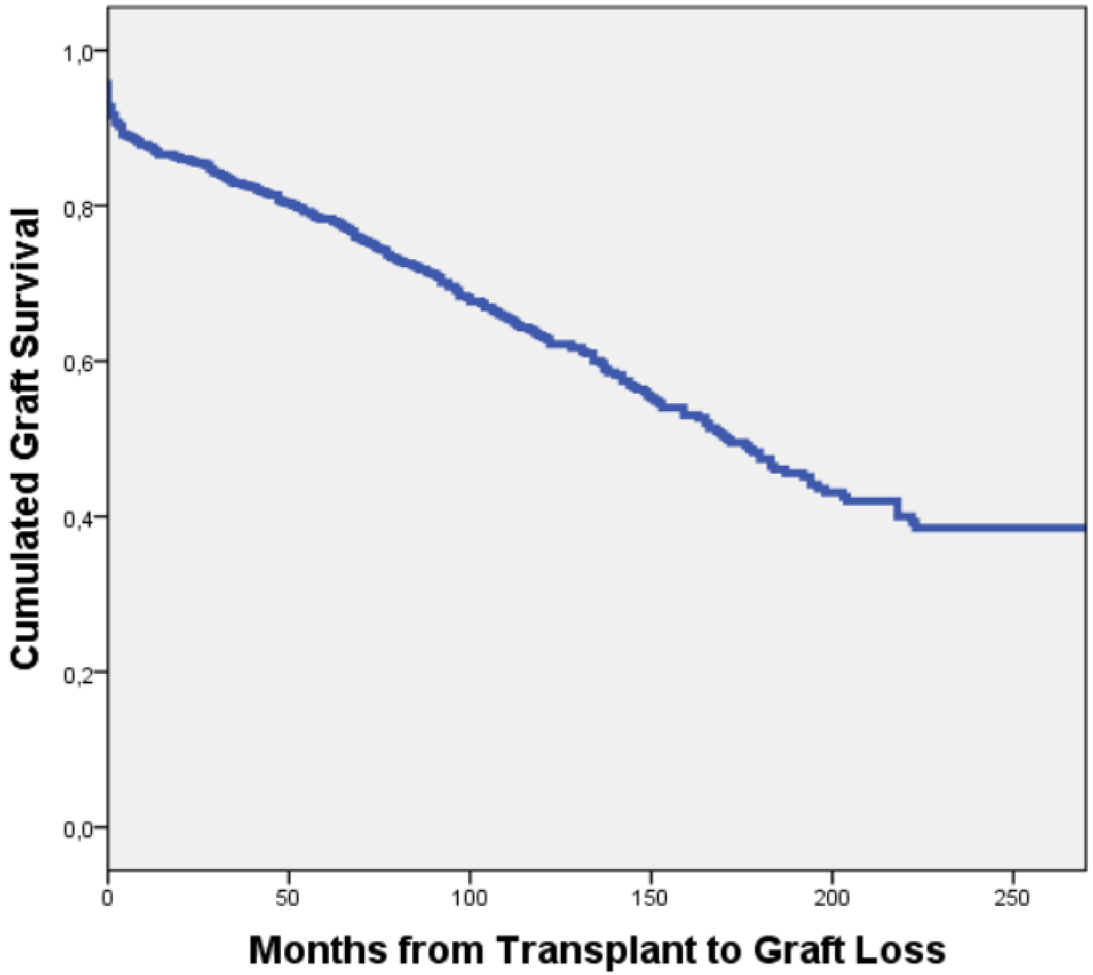

Cumulative death censored graft survival curve from the time of transplant of all kidney transplants performed in our institution in the study time (n=1098).

Global Graft Survival (n=1098) from the transplant

DCGS 12 months → 87.4%

DCGS 36 months → 82.6%

DCGS 60 months → 78.3%

DCGS 100 months → 68.2%

DCGS 150 months → 55.2%

DCGS 200 months → 43.1%

Comparative NP after 6 months vs. non NP patients

Log-rank, $p < 0.001$

Graft survival from transplant in patients with NP after 6 months (n=204)

DCGS 12 months → 98%

DCGS 36 months → 88.7%

DCGS 60 months → 78.1%

DCGS 100 months → 56.3%

DCGS 150 months → 33.9%

DCGS 200 months → 8.8%

Figure S2

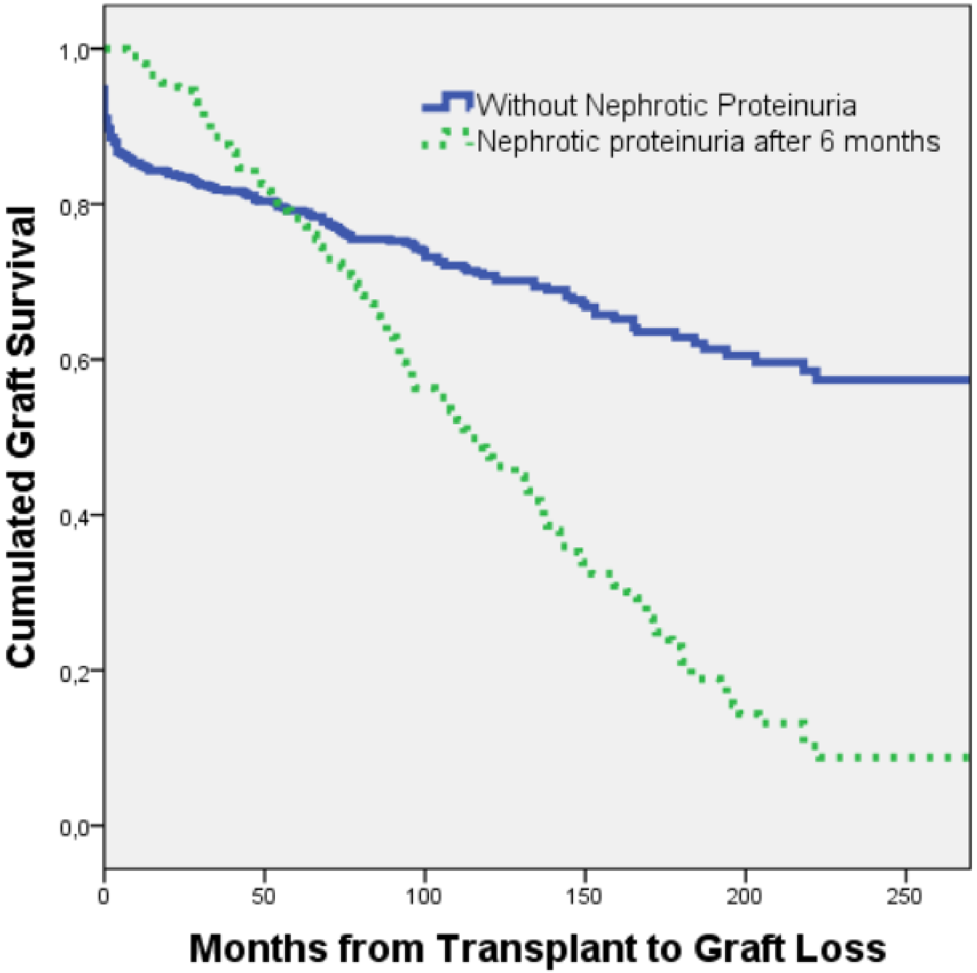

Death censored graft survival curve from the time of transplant. Continuous line represent recipients without nephrotic proteinuria (control group) and dashed line represents those with NP (study group)

Graft survival from transplant in patients without NP after 6 months (n=894)

DCGS 12 months → 84.9%

DCGS 36 months → 81.2%

DCGS 60 months → 79.1%

DCGS 100 months →73.2%

DCGS 150 months → 66.7%

DCGS 200 months → 60.5%

Figure S3

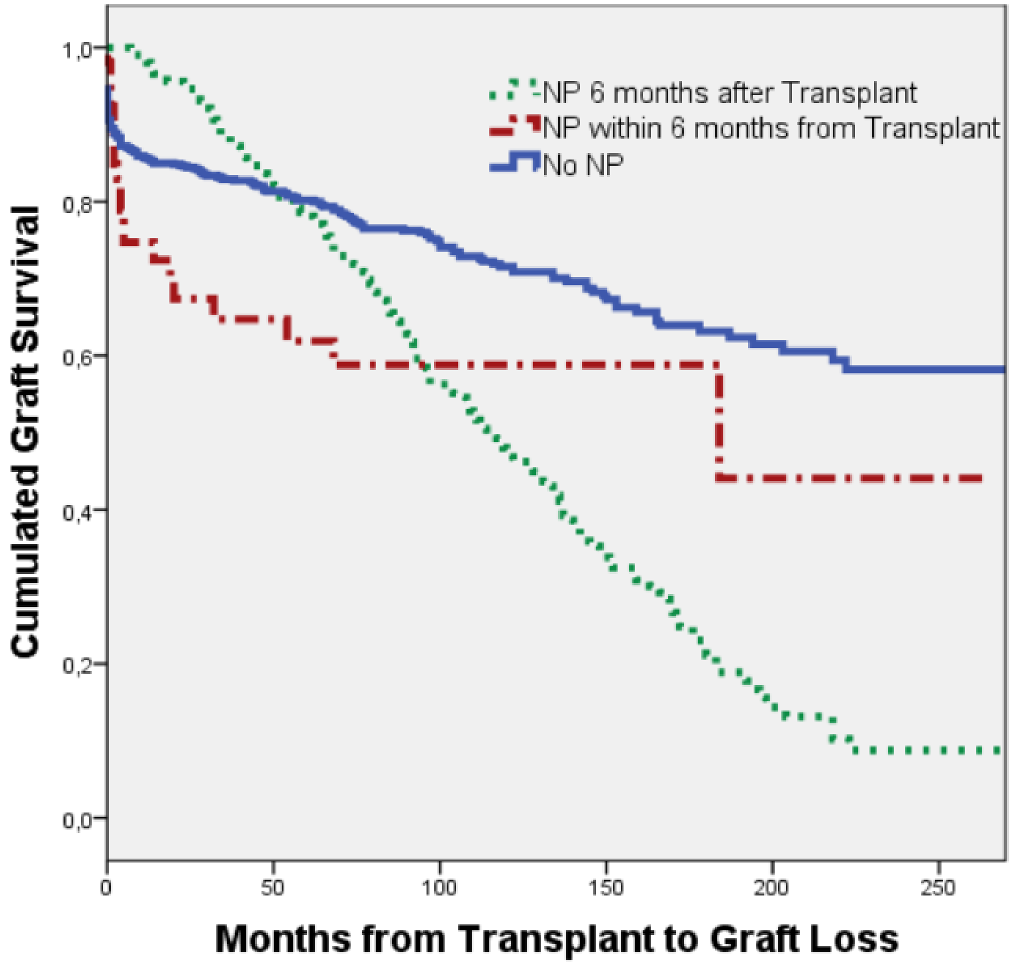

Death censored graft survival curve from the time of transplant. Blue line represent recipients without nephrotic proteinuria (control group), and green dashed line represents those with NP after 6 months and red dashed lines those with NP within 6 months.

Comparative NP after 6 months vs. NP within 6 months

Log-rank, p =0.907

Comparative NP within 6 months vs. non NP patients

Log-rank, p =0.015*

Graft survival from transplant in patients with NP within 6 months (n=53)

DCGS 12 months → 74.7%

DCGS 36 months → 58.8%

DCGS 60 months → 61.9%

DCGS 100 months → 58.8%

DCGS 150 months → 58.8%

DCGS 200 months → 44.1%
